# Midregional proatrial naturetic peptide (MRproANP) and copeptin (COPAVP) as predictors of all-cause mortality in recently diagnosed mild to moderate COPD—results from COSYCONET

**DOI:** 10.1186/s12931-024-02690-9

**Published:** 2024-01-24

**Authors:** S. Fähndrich, C. Herr, S. Teuteberg, P. Alter, S. Söhler, D. Soriano, J. Classen, J. Adams, V. Weinhold, H. Watz, B. Waschki, T. Zeller, M. Eichenlaub, F. C. Trudzinski, J. D. Michels, A. Omlor, F. Seiler, I. Moneke, F. Biertz, D. Stolz, T. Welte, H. U. Kauczor, K. Kahnert, R. A. Jörres, C. F. Vogelmeier, R. Bals, the German COSYCONET Cohort, Stefan Andreas, Peter Alter, Robert Bals, Jürgen Behr, Kathrin Kahnert, Thomas Bahmer, Burkhard Bewig, Ralf Ewert, Beate Stubbe, Joachim H Ficker, Christian Grohé, Matthias Held, Markus Henke, Felix Herth, Anne-Marie Kirsten, Henrik Watz, Rembert Koczulla, Juliane Kronsbein, Cornelia Kropf-Sanchen, Christian Herzmann, Michael Pfeifer, Winfried J Randerath, Werner Seeger, Michael Studnicka, Christian Taube, Hartmut Timmermann, Bernd Schmeck, Claus Vogelmeier, Tobias Welte, Hubert Wirtz

**Affiliations:** 1 Department of Pneumology, Faculty of Medicine, Medical Center, University of Freiburg, Killianstrasse 5, 79106 Freiburg, Germany; 2 Department of Internal Medicine V - Pulmonology, Allergology, Critical Care Care Medicine, Saarland University Hospital, Homburg, Germany; 3 Department of Medicine, Pulmonary, Critical Care and Sleep Medicine, Philipps University of Marburg (UMR), German Center for Lung Research (DZL), Marburg, Germany; 4 Airway Research Center North (ARCN), Pulmonary Research Institute at LungenClinic Grosshansdorf, Grosshansdorf, DZ Germany; 5 LungenClinic Grosshansdorf, Member of the German Center for Lung Research (DZL), Airway Research Center North (ARCN), Grosshansdorf, Germany; 6 Pneumology, Hospital Itzehoe, Itzehoe, Germany; 7 University Heart & Vascular Center Hamburg, Department of Cardiology, University Medical Center Hamburg-Eppendorf, Hamburg, Germany; 8 German Center for Cardiovascular Research (DZHK), Partner Site Hamburg/Kiel/Lübeck, Hamburg, Germany; 9 Department of Cardiology and Angiology, Medical Center, University of Freiburg, Freiburg, Germany; 10 Department of Pneumology and Critical Care, Heidelberg, Translational Lung Research Center Heidelberg (TLRC-H), Member of the German Center for Lung Research (DZL), Thoraxklinik Heidelberg gGmbH, Heidelberg, Germany; 11 Department of Thoracic Surgery, Faculty of Medicine, Medical Center-University of Freiburg, University of Freiburg, Freiburg, Germany; 12 Institute for Biostatistics, Hannover Medical School, Hannover, Germany; 13 Department of Respiratory Medicine, Research in Endstage and Obstructive Lung Disease Hannover (BREATH), Hannover, Member of the German Center for Lung Research (DZL), Hannover Medical School, Hannover, Germany; 14 Diagnostic and Interventional Radiology, Member of the German Center of Lung Research, University Hospital Heidelberg, Heidelberg, Germany; 15 Department of Internal Medicine V, Comprehensive Pneumology Center, Member of the German Center for Lung Research (DZL), LMU University Hospital, LMU Munich, Ludwig-Maximilians-University Munich (LMU), Munich, Germany; 16 Institute and Outpatient Clinic for Occupational, Social and Environmental Medicine, Munich, Germany; 17 Helmholtz Institute for Pharmaceutical Research Saarland (HIPS), Helmholtz Centre for Infection Research (HZI), Saarland University Campus, Saarbrücken, Germany

**Keywords:** COPD, Mortality, MRproADM, COPAVP, MRproANP, Prospective multicenter cohort study, COSYCONET

## Abstract

**Background:**

MRproANP and COPAVP are prognostic markers for mortality in chronic obstructive pulmonary disease (COPD). Furthermore, these biomarkers predict mortality due to cardiovascular diseases, which are important prognostically determining comorbidities in patients with COPD. However, less is known about these biomarkers in recently diagnosed mild to moderate COPD. Therefore, we analyzed these biomarkers as potential predictors of mortality in recently diagnosed mild to moderate COPD.

**Methods:**

The blood biomarkers considered were copeptin (COPAVP), midregional adrenomedullin (MRproADM), midregional proatrial naturetic peptide (MRproANP), and fibrinogen. Analyses were performed in patients with stable “recently diagnosed mild to moderate COPD” defined by GOLD grades 0–2 and diagnosis of COPD ≤ 5 years prior to inclusion into the COSYCONET cohort (COPD and Systemic Consequences—Comorbidities Network), using Cox regression analysis with stepwise adjustment for multiple COPD characteristics, comorbidities, troponin and NT-proBNP.

**Results:**

655 patients with recently diagnosed mild to moderate COPD were included. In the initial regression model, 43 of 655 patients died during the 6-year follow-up, in the final model 27 of 487. Regression analyses with adjustment for confounders identified COPAVP and MRproANP as statistically robust biomarkers (p < 0.05 each) of all-cause mortality, while MRproADM and fibrinogen were not. The fourth quartile of MRproANP (97 pmol/L) was associated with a hazard ratio of 4.5 (95%CI: 1.6; 12.8), and the fourth quartile of COPAVP (9.2 pmol/L) with 3.0 (1.1; 8.0). The results for MRproANP were confirmed in the total cohort of grade 0–4 (n = 1470 finally).

**Conclusion:**

In patients with recently diagnosed mild to moderate COPD, elevated values of COPVP and in particular MRproANP were robust, independent biomarkers for all-cause mortality risk after adjustment for multiple other factors. This suggests that these markers might be considered in the risk assessment of early COPD.

**Supplementary Information:**

The online version contains supplementary material available at 10.1186/s12931-024-02690-9.

## Introduction

COPD is a multi-system disease caused by the interaction of inhaled compounds, genetic predisposition, and environmental and socioeconomic factors [1]. When diagnosed at to moderate COPD (Global Initiative for Chronic Obstructive Lung Disease [GOLD] stage 0–2), it is difficult for the physician to identify patients at increased risk of death, as forced expiratory volume (FEV_1_) spirometry is a poor predictor of mortality even in severe COPD [2]. In order to be able to focus diagnostics and therapy on at-risk patients, there is a need to find better biomarkers for risk assessment. In this paper, we analyzed biomarkers that are also associated with cardiovascular diseases [3, 4], since COPD is a systemic disease whose prognosis also depends on the severity of the (especially cardiovascular) comorbidities [5].

So far, the markers copeptin (COPAVP) [6], midregional adrenomedullin (MRproADM) [7], midregional proatrial naturetic peptide [8] and fibrinogen [9] that can be measured in blood, have been examined primarily in patients with advanced COPD with regard to mortality risk [6, 8, 10]. COPAVP is a cleavage product of the vasopressin precursor peptide [11], MRproADM acts as a pleiotropic mediator in response to inflammatory, vascular, and metabolic stimuli in various tissues, including the lung [12], and MRproANP is the product of the cleaved precursor proANP that is secreted by right atrial ventricular cardiomyocytes in response to fluid overload or mechanical overexpansion [13]. These markers are attributed to cardiovascular diseases under stable conditions [12, 14, 15], as well as sepsis [12, 16, 17]. Fibrinogen is currently accepted by the US Food and Drug Administration (FDA) and the European Medicines Agency (EMA) for COPD studies [18] and known to be associated with exacerbation risk, COPD-related hospitalizations and mortality [18].

So far, however, it has not been investigated whether these biomarkers also predict all-cause mortality in patients with mild to moderate COPD (GOLD grades up to 2) who have been diagnosed recently (≤ 5 years). In these early stages it is difficult to identify patients who may need intensified screening. We thus examined COPAVP, MRproADM, MRproANP and fibrinogen specifically in patients with non-severe and recently diagnosed COPD, including patients of all COPD severities for comparison. The analysis was based on data from COSYCONET (COPD and Systemic Consequences–Comorbidities Network) [19], which is a large cohort of well-characterized individuals with stable COPD of all grades. In addition to the four biomarkers mentioned, we accounted for a broad panel of COPD-related risk factors, comorbidities and other biomarkers to determine mortality risk over a 6-year follow-up period.

## Methods

### Study design and patients

This longitudinal observational analysis was based on data from 2741 patients with stable COPD from the COSYCONET cohort of GOLD grades 0 to 4; see Clinical-Trials.gov with identifier NCT01245933 [19]. The study was approved by the ethics committees of all study sites, performed according to the declaration of Helsinki, and all patients gave their written informed consent. Routine laboratory parameters including fibrinogen, creatinine, c-reactive protein (CRP), HbA1c [20] were analyzed in all study sites, while the concentrations of COPAVP, MRproADM and MRproANP in P100-stabilized plasma were assessed on a Kryptor Compact Plus (BRAHMS GmbH, Hennigsdorf, Germany) in the central biobank (Homburg/Saarland). B-type natriuretic peptide (NT-proBNP) and troponin I were measured in the biobank (MILLIPLEX, Merck Millipore, Darmstadt, Germany) and the University Heart Center Hamburg, Germany (Architect STAT, Abbott Diagnostics, Wiesbaden, Germany), respectively.

All clinical and functional assessments have been previously described [19]. These included the assessment of age, body mass index (BMI), smoking status and pack years, forced expiratory volume in 1 s (FEV_1_), forced vital capacity (FVC), lung diffusing capacity for carbon monoxide (TLCO), ankle-brachial index (ABI), left-ventricular ejection fraction from echocardiography (LVEF) (for methodology see Alter et al. [21–23]), 6-min walk distance (6-MWD), arterial PaO_2_ and PaCO_2_ from the earlobe, the St George’s Respiratory Questionnaire (SGRQ), the number of moderate to severe exacerbations in the previous year, and patient-reported, physician-diagnosed comorbidities (hypertension, coronary artery disease, heart failure, history of myocardial infarction, history of stroke, hyperlipidemia, diabetes mellitus). Peripheral vascular dysfunction was defined as ABI ≤ 0.9, hypoxemia as PaO_2_ < 60 mmHg, and hypercapnia as PaCO_2_ > 55 mmHg. Predicted values for lung function were taken from GLI [24, 25].

Patients with a ratio FEV_1_/FVC < 0.7 were categorized as GOLD grades 1–4 according to FEV_1_ [26], while those with a ratio ≥ 0.7 were kept in the analysis and termed as “GOLD 0”, as this group had been turned out to be informative [23] and should be of special interest with regard to early COPD. Patients with mild to moderate COPD (GOLD grades up to 2) who had been diagnosed recently (≤ 5 years) were defined as “recently diagnosed mild to moderate COPD” group. Moreover, all patients were categorized as GOLD groups A/B/E based on their symptoms assessed by the mMRC and their exacerbation history [27]. However, groups and grades were not used in the association analyses.

The outcome was all-cause mortality (follow-up for observation time during the study, either to death or to study exclusion for other reasons, was taken into account) until the time of study visit 6 [6 years after inclusion]. If a patient missed one of the follow-up visits (6, 18, 36, 54, 72 months after inclusion), the research assistants ascertained survival status (and, in the case of death, date of death) by contacting relatives, GPs, and hospitals. When an exact date of death was not available, the date was attributed (assuming the 15th of the month if the month but not the date was known; midyear was assumed if only the year of last contact was known) [5, 20, 28].

### Statistical analyses

Median values and quartiles, or numbers and percentages are given for data description, depending on the type of variable. We calculated the risk of mortality associated with the biomarkers in a series of Cox proportional hazard regression models, adjusting stepwise for COPD characteristics, further established risk factors, and common cardiovascular diseases and biomarkers (for the single variables see Table 2 and Additional file 1: Table S1). This approach was chosen in order to determine the robustness of the association with the four biomarkers that were of primary interest. In order to handle their skewed distributions and derive useful cut-off values, we defined binary variables comprising their respective upper quartile versus the lower three quartiles. For other variables such as NT-proBNP, troponin I, HbA1c and CRP, a logarithmic transformation turned out to be sufficient to account for the skewed distributions. Statistical significance was assumed for p < 0.05. All analyses were performed using SPSS (Version 29, IBM, Armonk, NJ, USA).

## Results

**Fig. 1:**
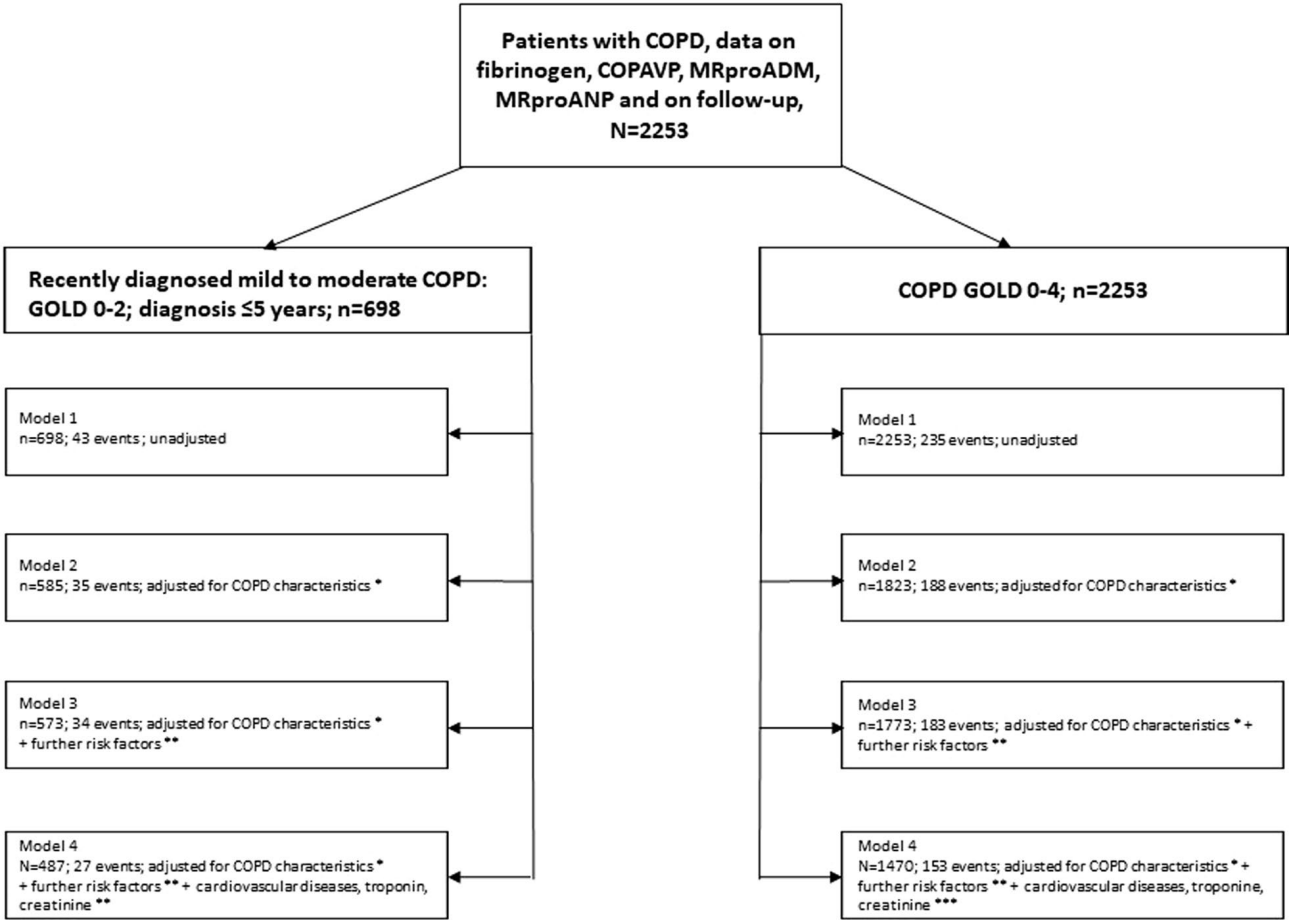
Flowchart of the sequential Cox proportional hazard regression analyses in the primary study group of early COPD (left branch) and the total population (right branch)

Of the 2741 patients, 1470 patients with COPD grades 0–4 had complete data on survival status, the biomarkers MRproADM, COPAVP, MRproANP, fibrinogen and all confounders used in the final regression analyses (Table 2). Of these patients, 153 died (Fig. 1). The sub-group with recently diagnosed mild to moderate COPD (grade 0–2 and diagnosed with COPD within 5 years prior to inclusion) and complete data comprised 487 patients, of whom 27 died (Fig. 1). Table 1 lists the characteristics of both groups of patients.

**Table 1:** Baseline characteristics of the patients with complete data to perform the final regression analyses shown in Table 2 and the corresponding Additional file 1: Table S1

	Recently diagnosed mild to moderate COPD, n = 487	GOLD 0–4 n = 1470
Demographics		
Age (years)	64 (58; 70)	65 (59; 70)
Men (%)	279 (57.3%)	898 (61.1%)
BMI (kg/m^2^)	27.1 (24.3; 31.1)	26.6 (23.7; 30.0)
Current smoker (%)	173 (35.5%)	430 (29.3%)
Packyears	42 (23; 64)	42 (21; 66)
Comorbidities		
Hypertension (%)	273 (56.1%)	846 (57.6%)
Diabetes mellitus (%)	73 (15.0%)	205 (13.9%)
Hyperlipidemia (%)	244 (50.1%)	668 (45.4%)
Coronary artery disease (%)	79 (16.2%)	249 (16.9%)
Heart failure (%)	23 (4.7%)	80 (5.4%)
History of myocardial infarction (%)	44 (9.0%)	127 (8.6%)
History of stroke (%)	27 (3.5%)	60 (4.1%)
COPD characteristics		
FEV_1_% predicted (4)	68.8(59.3; 81.1)	56.0 (42.4; 71.5)
TLCO % predicted (4)	67.7 (54.8; 80.4)	59.3 (44.0; 73.3)
SGRQ	33.8 (21.4; 47.4)	39.5 (27.5; 54.6)
Ankle-brachial index (ABI) ≤ 0.9 (%)	17 (3.5%)	65 (4.4%)
LVEF (%)	60 (56; 68)	60 (56; 67)
Moderate exac. in prev. year ≥ 1 (%)	131 (26.9%)	517 (35.2%)
6-min walk distance (6-MWD) (m)	466 (401; 512)	437 (369; 490)
Hypoxemia PaO_2_ < 60 mmHg (%)	68 (14.0%)	267 (18.8%)
Hypercapnia PaCO_2_ > 55 mmHg (%)	0 (0%)	2 (0.1%)
GOLD 0/1/2/3/4 (%)	24.4/14.4/61.2%	13.0/8.4/39.7/31.7/7.1%
GOLD A/B/E	56.3/16.8/26.9%	42.4/22.6/35.1%
Biomarkers from blood		
Glycated hemoglobin (mmol/mol)	5.80 (5.6; 6.2)	5.8 (5.5; 6.1)
Creatinine (mg/dL)	0.87 (0.75; 1.02)	0.86 (0.75; 1.00)
CRP (mg/dL)	0.40 (0.16; 0.63)	0.45 (0,20; 0.68)
NT-proBNP (pg/mL)	180.8 (35.9; 386.2)	189.3 (33.9; 379.5)
Troponin (pg/mL)	3.7 (2.3; 6.4)	3.7 (2.4; 6.5)
COPAVP (pmol/L)	5.29 (3.74; 8.20)	5.55 (3.76; 8.68)
MRproADM (nmol/L)	0.663 (0.566; 0.809)	0.675 (0.571; 0.814)
MR-proANP (pmol/L)	65.6 (47.0; 91.2)	65.5 (47.5; 93.8)
Fibrinogen (g/L)	2.40 (1.74; 3.22)	2.44 (1.80; 3.19)

Median values and quartiles (in parentheses) are given, or absolute numbers and percentages (in parentheses, except for GOLD categories for which only percentages are given)

*BMI* body mass index, *FEV1* forced expiratory volume in 1 s, *TLCO* lung diffusing capacity for carbon monoxide, *SGRQ* St George’s Respiratory Questionnaire, *LVEF* left-ventricular ejection fraction from echocardiography, PaO2 and *PaCO2* arterial partial pressures of oxygen and carbon dioxide, respectively, from the earlobe, *GOLD* Global Initiative for Chronic Obstructive Lung Disease, *CRP* C-reactive protein, *NT-proBNP* -type natriuretic peptide, *COVAP* copeptin, *MRproADM* midregional adrenomedullin, *MRproANP* midregional pro atrial naturetic peptide

**Table 2:** Results of consecutive Cox regression analyses for mortality in patients with GOLD 0–2 and COPD diagnosis no more than 5 years prior to inclusion

**Recently diagnosed mild to moderate COPD**	**COPAVP** HR for upper quartile (9.18 pmol/L)		**MRproADM** HR for upper quartile (0.824 nmol/L)		**MRproANP** HR for upper quartile (97.3 pmol/L)		**Fibrinogen** HR for upper quartile (3.22 g/L)	
	HR (95% CI)	P value	HR (95% CI)	P value	HR (95% CI)	P value	HR (95% CI)	P value
**Model 1** Unadjusted, only four biomarkers	1.921 (1.025; 3.601)	**0.042**	1.658 (0.861; 3.191)	0.130	3.684 (1.902; 7.136)	** < 0.001**	1.717 (0.929; 3.172)	0.085
**Model 2** Four biomarkers adjusted for COPD characteristics *	2.667 (1.306; 5.447)	**0.007**	0.758 (0.330; 1.739)	0.512	2.974 (1.364; 6.488)	**0.006**	1.354 (0.663; 2.768)	0.406
**Model 3** Four biomarkers adjusted for COPD characteristics + further risk factors **	2.339 (1.077; 5.079)	**0.032**	0.744 (0.305; 1.814)	0.515	3.053 (1.355; 6.881)	**0.007**	1.387 (0.667; 2.885)	0.381
**Model 4 final** Four biomarkers adjusted for COPD characteristics + further risk factors ** + cardiovascular diseases, troponin & creatinine ***	2.988 (1.111; 8.039)	**0.030**	1.222 (0.420; 3.556)	0.712	4.491 (1.577; 12.789)	**0.005**	1.030 (0.397; 2.674)	0.951

The significant* p*-values are in bold

Hazard ratios are given for the respective biomarkers in the upper quartile versus the three lower quartiles

^*^Age, BMI, FEV1% predicted, TLCO % predicted, SGRQ total score, 6-min walking distance, ≥ 1 moderate/severe exacerbations in the previous year, smoking status, packyears, hypercapnia (PaCO2 > 55 mmHg), hypoxemia (PaO2 < 60 mmHg)

^**^All variables of model 2 plus sex, hypertension, diabetes, hyperlipidemia, log HbA1c, and log CRP

^***^All variables of model 3 plus heart failure, coronary artery disease, history of myocardial infarction, history of stroke, ankle-brachial index ≤ 0.90, log NT-proBNP, left-ventricular ejection fraction from echocardiography, troponin, creatinine

The results of the stepwise adjusted Cox regression analyses for the recently diagnosed mild to moderate COPD (GOLD 0–2, diagnosis ≤ 5 years) are shown in Table 2, while the analogous results for all patients of grades 0–4 are given in the Supplementary Table 1.

### Patients with recently diagnosed mild to moderate COPD

Using the stepwise approach, in the recently diagnosed mild to moderate COPD group the following results were obtained. When including only the four selected biomarkers as predictors, COPAVP and MRproANP were significantly (p < 0.05) related to mortality but MRproADM and fibrinogen not (Model 1). Table 2 shows the respective hazard ratios and their 95% confidence intervals.

When introducing age, BMI, FEV_1_% predicted, TLCO % predicted, SGRQ total score, 6-min walking distance, the occurrence of ≥ 1 moderate/severe exacerbations in the previous year, smoking status, pack years, hypercapnia (pCO_2_ > 55 mmHg) and hypoxemia (pO_2_ < 60 mmHg) as further predictors (Model 2), COPAVP and MRproANP remained significant (p < 0.05 each). Among the covariates, only age (p = 0.012) and hypoxemia (p < 0.001) were significantly associated with mortality.

COPAVP and MRproANP were still significant (p < 0.05 each) when additionally sex, hypertension, diabetes, hyperlipidemia, log HbA1c, and log CRP were included as predictors (Model 3). Among the covariates, again only age (p = 0.027) and hypoxemia (p = 0.003) were significant.

When extending this set of predictors by inclusion of heart failure, coronary artery disease, history of myocardial infarction, history of stroke, ankle-brachial index ≤ 0.90, log NT-proBNP, troponin I, creatinine and the left-ventricular ejection fraction from echocardiography (Model 4), COPAVP and MRproANP remained significant (p < 0.05 each). In addition, age (p = 0.027), hypoxemia (p = 0.001), sex (p = 0.032), coronary artery disease (p = 0.050) and a history of myocardial infarction (p = 0.047) were significant. This sequence of Cox regression model demonstrated that COPAVP and in particular MRproANP were statistically independent and robust biomarkers of mortality risk. Noteworthy, the hazard ratios regarding the upper quartiles of COPAVP and MRproANP were as high as about 3.0 and 4.5, respectively (Table 2).

The analysis also took into account the confounders “PRISm = FEV1/FVC ≥ 0.7 after bronchodilation but impaired spirometry (FEV1 < 80% of reference, after bronchodilation). The results did not change qualitatively. For example, in the final model 4 comprising the full set of predictors, the p-value of COPAVP changed from 0.030 to 0.024 and that of MRproANP from 0.005 to 0.004, while MRproADM and Fibrinogen maintained p-values far greater than 0.5. In these analyses, PRISm itself was not significantly associated with mortality.

We performed additional analyzes cardiovascular medication. When introducing the intake of RAAS, ACE inhibitors and beta-blockers into the final model 4 in patients with recent diagnosis of mild to moderate COPD, COPAVP and MRproANP remained statistically significant (p = 0.026 and p = 0,011, respectively). When alternatively introducing the intake of LABA, LAMA and ICS, both markers were still significant (p = 0.031 and p = 0.006, respectively). This demonstrated that medication did not interfer with their predictive value regarding mortality risk.

### All patients with COPD

The analysis was performed in the same manner as for the subgroup with early COPD, and the results are given in the Additional file 1: Table S1. When using only the four biomarkers (Model 1), COPAVP, MRproADM and MRproANP were significant (p < 0.001 each). When extending the predictors to Model 2, MRproANP and fibrinogen were significant, and among the other variables age, BMI, FEV_1_% predicted, 6-MWD, smoking status and pack years (p < 0.05 each). In Model 3, MRproANP and fibrinogen remained significant, in addition to age, BMI, FEV_1_% predicted, 6-MWD, smoking status and hypertension (p < 0.05 each). In Model 4, again MRproANP and fibrinogen were significant predictors of mortality, and in addition age, BMI, FEV_1_% predicted, 6-MWD, smoking status, hypertension, sex, and ABI (p < 0.05 each). It should be noted that the hazard ratio for MRproANP was only 1.6 (95%CI 1.1; 2.4), similar to that of fibrinogen with 1.5 (1.0; 2.1), and thus markedly smaller than in the group of early COPD.

## Disscussion

Patients with COPD often show cardiovascular comorbidities that are a major cause of death in these patients [5, 20, 28–35]. The main finding of the present analysis was that, in addition to COPAVP, MRproANP was a robust predictor of all-cause mortality in patients recently diagnosed mild to moderate COPDdefined as at maximum moderate grade (GOLD 0–2) with a diagnosis at most 5 years prior to inclusion in the study. The robustness was reflected in the fact that both biomarkers maintained their significant association and the magnitude of their hazard ratios after adjustment for a multitude of other risk factors. Remarkably, MRproADM and fibrinogen showed no significant association with all-cause mortality in early COPD.

Results were slightly different in the total study population comprising COPD patients of all grades 0–4, since MRproANP was still a robust predictor but COPAVP not, while fibrinogen turned out to be also related to mortality. This indicated that the usefulness of the four biomarkers depended on the severity and history of COPD. The latter was suggested by the following observation. If only grade 0–2 was required and the requirement of a diagnosis of COPD ≤ 5 years prior to inclusion was omitted, MRproANP was still a significant predictor but COPAVP no more, similar to fibrinogen. This might indicate that COPAVP is relevant only at an early stage, while MRproANP is relevant at all severities and stages of the disease but especially useful in early COPD according to the magnitude of its hazard ratio.

Our results are partially consistent with those of other investigators who studied these biomarkers to predict mortality in all severities of COPD. We confirmed previous findings of MRproANP and COPAVP being predictors in COPD of severity up to grade 4 [36] but for MRproADM we found an association only for unadjusted data. Our data are in line with previous publications that analyzed MR-proANP as predictor for mortality in patients with COPD when they are admitted to the hospital for exacerbation [8]. As we aimed to compare the predictive value of the single markers, we did not combine them with known clinical risk scores such as the BODE (Body mass index, airflow Obstruction, Dyspnea) index [36] or the ADO (Age, Dyspnea, airflow Obstruction) index [37]. In line with the data from the COMIC study [38], fibrinogen was less powerful as marker for mortality risk in COPD and only of value if patients of high severity were included. In early COPD it had no predictive power compared with the more specific cardiovascular-like markers COPAVP and MRproANP. One of the reasons might be that fibrinogen plays a role in coagulation and, as an acute phase reactant, is therefore more associated with inflammation and exacerbations of COPD [39] which occurred more often in the total group that in the group of early COPD (see Table 1) and had an effect, although at the time of the study visits, COSYCONET patients were required to be in a stable clinical state. It also seems of interest that in presence of the four biomarkers tested and the other confounders neither NT-proBNP nor troponin were significantly associated with mortality risk.

In order to keep the analysis simple and to facilitate interpretation, we decided to use quartiles of the four biomarkers instead of complicated transformations that would deal with their highly skewed distributions. These quartiles were derived from the values of the total population in order to keep the analyses of both groups comparable and to avoid the need for a priori information on disease severity that would be needed otherwise. We also decided to compare the highest quartiles with the pooled other three quartiles in order to reduce the number of categories in the analyses and to preserve as much statistical power as possible. This was needed as the number of events (deaths) was only 43/27 in 655/487 patients of the initial/final model in the early COPD group, although we performed a follow-up until visit 6 of COSYCONET. Using this simple approach, the cut-off value relevant for MRproANP in the early COPD group was identified as about 97 pmol/L, and that of COPAVP as about 9.2 pmol/L. These values might be of practical use, if one or both of these markers are determined. We do not advocate at the present stage that these values should be routinely measured, as more data will be needed for such a conclusion. However, when designing the analysis, we had in mind the general practitioner who is most likely to encounter a newly diagnosed patient with mild to moderate COPD and often does not have the panel of instruments used by a specialist. If the aim is to identify high-risk patients who need more screening and medical care, a blood sample is the simplest approach.

The strengths of our analysis are the inclusion of a broad spectrum of confounders and a long follow-up period. Due to the large sample size, it was possible to define a group of patients with recently diagnosed mild to moderate COPDdefined as GOLD grade 0–2 with a recent (≤ 5years) diagnosis of COPD. To cover other risk factors than biomarkers as broad as possible, we included comorbidities as well as objective measurements of lung function, echocardiography and ankle-brachial index. A limitation of our study was that the biomarkers were determined only once at baseline. Therefore, we had no information about their time course which might be informative, too. Furthermore, similar to most COPD studies, the cause of death could not be determined in all patients, thus cardiovascular mortality could not be specifically addressed in addition to all-cause mortality. Another limitation is that we were only able to consider 27 deaths for the analysis in the group of newly diagnosed mild to moderate COPD. On the other hand, it should be considered that the results were remarkably robust.

In conclusion, MRproANP and COPAVP are statistically robust biomarkers for all-cause mortality risk in patients with stable early COPD that was defined as GOLD grade 0–2 and diagnosis within 5 years prior to inclusion. The association with MRproANP was confirmed when considering all patients of grades 0–4. This type of robustness across all grades might be an argument to include this biomarker in the risk assessment of early COPD.

### Supplementary Information

**Additional file 1: Table S1.** Results of consecutive Cox regression analyses in all patients of grades GOLD 0-4. Hazard ratios are given for the respective biomarkers in the upper quartile versus the three lower quartiles. * Age, BMI, FEV_1_% predicted, TLCO % predicted, SGRQ total score, 6-minute walking distance, ≥1 moderate/severe exacerbations in the previous year, smoking status, pack years, hypercapnia (PaCO_2_ >55 mmHg), hypoxemia (PaO_2_ <60 mmHg). ** All variables of model 2 plus sex, hypertension, diabetes, hyperlipidemia, log HbA1c, and log CRP. *** All variables of model 3 plus heart failure, coronary artery disease, history of myocardial infarction, history of stroke, ankle-brachial index ≤0.90, log NT-proBNP, left-ventricular ejection fraction from echocardiography.

## Data Availability

COSYCONET is an ongoing, long-term, multi-center observational study the data of which are not intended to be available without demand. If there is interest in the analysis of specific questions, however, there is a formalized procedure for submitting an application to the study office, which will be evaluated by the steering committee on scientific grounds. There is no limitation for this application except proven expertise in COPD studies.
