# Supplementary material for: Midregional proatrial naturetic peptide (MRproANP) and copeptin (COPAVP) as predictors of all-cause mortality in recently diagnosed mild to moderate COPD—results from COSYCONET
Source: Respir Res. 2024 Jan 24;25:56. doi: 10.1186/s12931-024-02690-9 (PMC10809634; doi:10.1186/s12931-024-02690-9)
Supplement: Supplementary file 1 — Additional file 1: Table S1. Results of consecutive Cox regression analyses in all patients of grades GOLD 0-4. Hazard ratios are given for the respective biomarkers in the upper quartile versus the three lower quartiles. * Age, BMI, FEV1% predicted, TLCO % predicted, SGRQ total score, 6-minute walking distance, ≥1 moderate/severe exacerbations in the previous year, smoking status, pack years, hypercapnia (PaCO2 >55 mmHg), hypoxemia (PaO2 <60 mmHg). ** All variables of model 2 plus sex, hypertension, diabetes, hyperlipidemia, log HbA1c, and log CRP. *** All variables of model 3 plus heart failure, coronary artery disease, history of myocardial infarction, history of stroke, ankle-brachial index ≤0.90, log NT-proBNP, left-ventricular ejection fraction from echocardiography. [file 12931_2024_2690_MOESM1_ESM.docx]

| **Patients of grades GOLD 0-4** | **COPAVP**  HR for upper quartile  (9.18 pmol/L) | | **MRproADM**  HR for upper quartile  (0.824 nmol/L) | | **MRproANP**  HR for upper quartile  (97.3 pmol/L) | | **Fibrinogen**  HR for upper quartile  (3.22 g/L) | |  |
| --- | --- | --- | --- | --- | --- | --- | --- | --- | --- |
|  | HR (95% CI) | P value | HR (95% CI) | P value | HR (95% CI) | P value | HR (95% CI) | P value |  |
| **Model 1**  Unadjusted, only four biomarkers | 1.848  (1.403; 2.435) | **<0.001** | 1.915  (1.423; 2.577) | **<0.001** | 1.851  (1.390; 2.465) | **<0.001** | 1.283  (0.970; 1.696) | 0.081 |  |
| **Model 2**  Four biomarkers adjusted for COPD characteristics * | 1.337  (0.971; 1.841) | 0.075 | 1.183  (0.817; 1.713) | 0.374 | 1.755  (1.243; 2.479) | **0.001** | 1.378  (1.007; 1.885) | **0.045** |  |
| **Model 3**  Four biomarkers adjusted for COPD characteristics  + further risk factors ** | 1.198  (0.855; 1.678) | 0.295 | 1.166  (0.800; 1.699) | 0.425 | 1.769  (1.246 (2.511) | **0.001** | 1.417  (1.031; 1.948) | **0.032** |  |
| **Model 4 final**  Four biomarkers adjusted for COPD characteristics + further risk factors ** + cardiovascular diseases, troponin & creatinine *** | 1.334  (0.908; 1.958) | 0.142 | 1.259  (0.817; 1.939) | 0.297 | 1.605  (1.065; 2.419) | **0.024** | 1.473  (1.041; 2.085) | **0.029** |  |

**Supplementary Table 1:** Results of consecutive Cox regression analyses in all patients of grades GOLD 0-4. Hazard ratios are given for the respective biomarkers in the upper quartile versus the three lower quartiles. * Age, BMI, FEV_1_ % predicted, TLCO % predicted, SGRQ total score, 6-minute walking distance, ≥1 moderate/severe exacerbations in the previous year, smoking status, packyears, hypercapnia (PaCO_2_ >55 mmHg), hypoxemia (PaO_2_ <60 mmHg). ** All variables of model 2 plus sex, hypertension, diabetes, hyperlipidemia, log HbA1c, and log CRP. *** All variables of model 3 plus heart failure, coronary artery disease, history of myocardial infarction, history of stroke, ankle-brachial index ≤0.90, log NT-proBNP, left-ventricular ejection fraction from echocardiography, troponin, creatinine
